# Performance Evaluation of a Rapid Antigen Test (RAT) during Omicron Pandemic Wave in Greece, Conducted by Different Personnel, and Comparison with Performance in Previous Wave (Alpha Variant) Period

**DOI:** 10.3390/diagnostics12051048

**Published:** 2022-04-21

**Authors:** Maria A. Kyritsi, Matthaios Speletas, Varvara Mouchtouri, Evangelia Vachtsioli, Dimitrios Babalis, Olympia Kouliou, Anastasia Tsispara, Maria Tseroni, Christos Hadjichristodoulou

**Affiliations:** 1 Laboratory of Hygiene and Epidemiology, Faculty of Medicine, University of Thessaly, 41222 Larissa, Greece; mkiritsi@uth.gr (M.A.K.); mouchtourib@med.uth.gr (V.M.); evaxtsioh@yahoo.gr (E.V.); 2 Department of Immunology and Histocompatibility, Faculty of Medicine, University of Thessaly, 41500 Larissa, Greece; maspel@med.uth.gr; 3 Emergency Department, General Hospital of Larissa, 41221 Larissa, Greece; dbabales@yahoo.com (D.B.); olympia.kouliou@hotmail.com (O.K.); tsispara@gmail.com (A.T.); 4 Directorate of Epidemiological Surveillance of Infectious Diseases, National Public Health Organization, 15123 Athens, Greece; m.tseroni@eody.gov.gr

**Keywords:** SARS-CoV-2, Omicron variant, rapid antigen test, evaluation

## Abstract

Due to the prevailing ambiguity regarding the performance of rapid antigen tests (RATs) for B.1.1.529 (Omicron) variant diagnosis, a commercial RAT was evaluated in the emergency ward of a general hospital in Larissa, Central Greece. The sampling and the evaluation were repeated twice by different personnel. Discordance between the two samplings was observed regarding the sensitivity (47.5%, 95% CI: 39.0–56.1 vs. 78.6%, 95% CI: 69.1–86.2) and specificity (93.8%, 95% CI: 86.0–97.9 vs. 100.0%, 95% CI: 93.3–100.0) of the RAT. Furthermore, the test displayed slightly lower sensitivity (78.6% vs. 85.5%, 95% CI: 79.1–90.5) compared to its initial evaluation that was conducted by our team when the B.1.1.7 (Alpha) variant was dominant.

## 1. Introduction

It has been almost two years since the coronavirus disease (COVID-19), caused by severe acute respiratory syndrome coronavirus 2 (SARS-CoV-2), was declared by the World Health Organization (WHO) as a major public health emergency worldwide [1]. Most countries under the guidance of the scientific community have taken a lot of measures to prevent the COVID-19 spread, especially in populations vulnerable to developing severe disease or even death, with the ultimate purpose being to prevent health systems becoming overloaded. Unfortunately, SARS-CoV-2 infection proved to vary in clinical manifestations, from asymptomatic, yet contagious, to severe acute respiratory distress syndrome [2], which according to WHO data, resulted in 437,333,859 confirmed cases of COVID-19, including 5,960,972 deaths, as of 2 March 2022 [3].

In this context, one of the main pillars of SARS-CoV-2 surveillance was the mass screening policy, in order to achieve a quick and reliable diagnosis of infected persons and implement infection control measures aiming to limit the virus spread. Real-time reverse transcription polymerase chain reaction (RT-PCR) has been proposed as the gold standard method for SARS-CoV-2 detection by the WHO and CDC [4,5] and was the only diagnostic method used for screening before the implementation of rapid antigen diagnostic tests (RATs). Although highly sensitive for diagnosing COVID-19, RT-PCR and Nucleic Acid Amplification Test (NAAT) methods (e.g., isothermal amplification-based methods and high-throughput sequencing) are expensive, time consuming and require technical expertise and laboratory capacity [6]. In addition, the ongoing pandemic and the growing demand for molecular testing have resulted in an urgent need for the development of RATs for frontline testing and diagnosing SARS-CoV-2 infection. RATs are relatively inexpensive, simple to perform and give results within a few minutes [6], thus providing the ability to greatly increase both the access to and speed of testing.

Soon after the onset of the pandemic, many RATs were developed that meet the World Health Organization (WHO) requirements for COVID-19 diagnosis [7]. Nevertheless, they have presented important diagnostic evaluation challenges due to the emerging SARS-CoV-2 variants [8]. Usually, new evidence on variants is acquired through epidemic intelligence, rules-based genomic variant screening, or other scientific sources. If there is clear evidence that indicates a significant impact on transmissibility, severity and/or immunity (i.e., that the emerging variant poses an increased risk to public health), it is characterized as a variant of interest (VOI) or variant of concern (VOC), with the aim being to prioritize global monitoring and research, and adapt accordingly the ongoing response to the COVID-19 pandemic. So far, five variants have been designated as variants of concern (VOC): the B.1.1.7 (Alpha) variant, B.1.351 (Beta) variant, P.1 (Gamma) variant, B.1.617.2 (Delta) variant and the recently emerged B.1.1.529 (Omicron) variant [9]. The B.1.1.529 variant was reported to the WHO from South Africa on 24 November 2021 and was named the “Omicron” variant [10,11]. The Omicron variant has some deletions and significantly more mutations (above 30) than previous SARS-CoV-2 variants, particularly in the gene that encodes the spike protein (S-gene) [12].

As new variants of SARS-CoV-2 emerge, it is clear that changes in the viral genome can result in changes to viral proteins and, therefore, the performance of the tests could be impacted [13]. In the present study, the diagnostic accuracy of a rapid point-of-care antigen test (Rapid Test Ag 2019-nCoV-PROGNOSIS, BIOTECH, Larissa, Greece) was evaluated for the period that the Omicron variant was dominant in the city of Larissa, Greece. The same RAT was evaluated when the Alpha variant was dominant in Larissa and the results concerning its performance were compared [14]. Furthermore, two separate samplings by different personnel were conducted to determine the reproducibility of the test.

## 2. Materials and Methods

In order to evaluate the performance of the rapid test, a sampling on 30 December 2021 was conducted by hospital personnel. Clinical samples were collected at the Emergency ward of the General Hospital of Larissa from early symptomatic patients (presenting with a fever and/or cough and/or headache within 5–7 days) or asymptomatic persons reporting close contact to confirmed COVID-19 cases, as part of the routine diagnostic procedure. In detail, two nasopharyngeal swabs from 219 individuals (all aged > 18 years) were obtained simultaneously—the first from one nostril for molecular analysis, and the second from the other nostril according to manufacturer’s specifications for antigen testing.

Detection of SARS-CoV-2 antigens was performed with Rapid Test Ag 2019-nCov (PROGNOSIS, BIOTECH, Larissa, Greece) which is a qualitative, lateral flow immunoassay designed to detect the presence or absence of nucleocapsid protein (N) of SARS-CoV-2 in nasal or nasopharyngeal swab specimens that are directly collected, strictly under the manufacturer’s recommendations.

Concerning the molecular analysis, RNA extraction was performed on a KingFisher Flex System (ThermoFisher Scientific, Waltham, MA, USA) using the MagMAX™ Viral/Pathogen Nucleic Acid Isolation Kit (Applied Biosystems™, Waltham, MA, USA). For the RT-PCR, the TaqPath™ COVID-19 CE-IVD RT-PCR Kit (Applied Biosystems™, Waltham, MA, USA), which targets three different SARS-CoV-2 specific genomic regions (Gene ORF1ab, N Protein and S Protein) was used following the manufacturer’s instructions, on a validated QuantStudio™ 5 Real-Time PCR System (ThermoFisher Scientific, Waltham, MA, USA).

With the TaqPath™ COVID-19 CE-IVD RT-PCR Kit, when the sample is positive for the Omicron variant, the S-target gene is not detected because the genome contains the spike deletion at position 69–70 (called S gene dropout or S gene target failure—SGTF) and this test was used as a marker for the variant [15]. S-gene dropout is typically not observed in the Delta variant [12,15].

After the results of the analyses, sensitivity (Se) and specificity (Sp) with a 95% confidence interval (95% CI) were estimated based on binomial distribution. The statistical analysis was performed using Excel, (Microsoft, Redmond, Washington, DC, USA) and IBM SPSS (version 25) (Microsoft, Redmond, Washington, DC, USA).

After obtaining the results of this specific evaluation, we decided to perform a second sampling conducted by laboratory personnel on 5 January 2022, and 151 samples were collected and analyzed following the same procedure as described above.

In addition, of the 201 RT-PCR positive samples with SGTF, 124 were randomly selected and sent for Next Generation Whole Genome Sequencing at the Biomedical Research Foundation, Academy of Athens. All 124 samples were confirmed as the B.1.1.529 (Omicron) variant.

## 3. Results

First sampling (30 December 2021): As mentioned in the Material and Methods section, this sampling was performed by personnel of a general hospital in Larissa. The hospital personnel (nurses and emergency room physicians), in addition to performing the RAT, were also responsible for conducting all the other diagnostic and therapeutic procedures that were required in the emergency ward. The molecular analysis showed that 139 samples were positive while 80 were negative for SARS-CoV-2. Sixty-six (66) out of 139 RT-PCR confirmed positive samples were also positive with the RAT. The method displayed a total sensitivity of 47.5% (66/139) (95% CI: 39.0–56.1). Concerning the specificity of the test, 75 out of 80 RT-PCR confirmed negative samples were also negative with the RAT, which corresponds to 93.8% specificity (75/80) (95% CI: 86.0–97.9). For samples with SGTF, the test displayed a sensitivity of 46.0% (51/111) (95% CI: 36.5–55.7) and without SGTF the sensitivity of the test was 53.6% (15/28) (95% CI: 33.9–72.5). Finally, for samples with SGTF and Ct ≤ 25, the sensitivity was 50.2% (49/97) (95% CI: 40.2–60.8).

Second sampling (5 January 2022): This sampling was performed by laboratory personnel (biotechnicians of the Laboratory of Hygiene and Epidemiology) whose sole task was to conduct the rapid antigen test. The molecular analysis of the samples identified 98 samples positive for SARS-CoV-2 and 53 that were negative. Seventy-seven (77) out of 98 RT-PCR positive samples were also positive for SARS-CoV-2 antigen detection and the total sensitivity was estimated to be 78.6% (77/98) (95% CI: 69.1–86.2). Concerning the specificity of the test, no false positive results were observed, which corresponds to 100.0% specificity (53/53) (95% CI: 93.3–100.0). For samples with SGTF, the sensitivity was 78.9% (71/90) (95% CI: 69.0-86.8%). For samples with SGTF and Ct ≤ 25, the sensitivity of the test was 91.7% (66/72) (95% CI: 82.7–96.9). Due to the low number of SGTF-negative samples (only eight) during the sampling on 5 January, the sensitivity for this group was not calculated.

For each Ct group, the number of samples, the calculations with nominator/denominator values and the sensitivity and specificity are shown in detail in Table 1.

## 4. Discussion

Although qRT-PCR is the gold standard method for COVID-19 diagnosis [16], RATs have been widely used for faster and low-cost mass screening [8]. It is believed that RATs have a greater impact on public health than NAATs for COVID-19 diagnosis and surveillance, since they can be undertaken locally, avoiding the need for centralized and reference testing laboratories that are difficult to organize, especially in low- and middle-income countries.

As new virus variants emerge, there is always the fear that a great number of genomic mutations, as observed in the Omicron variant, could affect the properties of certain viruses, such as transmissibility to humans, the infection severity, the immunity raised by vaccines, potential therapeutic regimens, and perhaps the ability of the existing diagnostic tools to accurately detect the virus [17]. Under this context, even though most genome changes have little to no impact on the properties of a virus, complementary evaluations of commercial RATs should be considered to find out if their performance is affected [17].

In the emergency ward of Greek hospitals, RATs are used as a point of care test and are usually conducted by hospital personnel (nurses or physicians). On the other hand, the personnel of the diagnostic laboratories (biotechnicians, biochemists and microbiologists) are responsible for the molecular analysis of the samples and not for the sampling process.

Our team evaluated a widely used RAT in Greece, Rapid Test Ag 2019-nCoV-PROGNOSIS, BIOTECH, over March and April 2021 and found a sensitivity of 85.5% (95% CI: 79.1–90.5) (for Ct values < 30 it reached 90.4%) and 99.8% specificity (95% CI: 98.8–100.0) (Table 1) [14]. At that time, the B.1.1.7 (Alpha) variant was dominant in Larissa (approximately 3500 cases per day) and the flow of patients in the emergency department was relatively low in comparison with the patient flow during the Omicron wave (approximately 50,000 cases per day). The hospital personnel could dedicate the required amount of time to conduct the RATs and at the same time perform all the other diagnostic and therapeutic procedures.

In the present study, our aim was to find out if the test was affected by the virus mutations, and more specifically by the B1.1.529 (Omicron) variant, and to what extent. Using the data acquired from the first sampling (30 December 2021), we concluded that the ability of the test to detect COVID-19 was significantly reduced since the sensitivity of the test fell to 47.5% from 85.5% (Table 1). To our surprise, the test gave five false positive results that were not in accordance with our previous findings (specificity 93.8% vs. 99.8%). This particular result in combination with the dramatic increase in the patients who visited the emergency department led us to the decision to conduct a new sampling performed exclusively by laboratory personnel (biotechnicians).

The results of the second sampling (5 January 2022) were significantly different. The sensitivity was slightly inferior to our initial evaluation (78.9% vs. 85.5%), while specificity reached 100.0%. The difference that was observed at the sensitivity of the test between the second sampling and the initial evaluation, was not statistically significant since the calculated RR was 1.08 with a 95% CI of 0.9–1.2, showing that the test exhibits a satisfactory performance towards the Omicron variant (Table 1).

So far, a few studies have been conducted that provide data on RAT performance concerning the Omicron variant. The U.S. Food and Drug Administration (FDA) in collaboration with the National Institutes of Health’s (NIH) Rapid Acceleration of Diagnostics (RADx) program conducted initial laboratory tests using heat-inactivated samples for some commercial antigen tests, which detected the Omicron variant, with a similar performance when detecting other variants [18]. Adamson et al. found that rapid antigen tests lagged in their ability to detect COVID-19 during an early period of disease using an occupational case cohort of 30 individuals with daily testing during an Omicron outbreak [19]. In our study, the number of the patients used for the evaluation was significantly higher (219 patients during the first sampling and 151 patients during the second sampling) and two different samplings were performed to acquire more robust results.

Our evaluation found that the specific RAT used for this study does not dramatically change performance characteristics concerning the B1.1.529 (Omicron) variant. Even though this finding is particularly encouraging, due to the absence of data it is not possible to extend the conclusions to the performance of rapid antigen diagnostics tests in general.

The present study outlines that careful evaluations must be performed and except of the of usual laboratory quality control, all variables that could affect a test’s performance, for example personnel experience and workload, should be taken into consideration. During the evaluation of the test, the severity of the epidemic wave caused by the Omicron variant (up to 50,000 cases per day in Greece) affected the availability of hospital personnel who were already working under adverse conditions (excessive workload, wearing protective equipment, etc.). The present wave demanded further expansion of testing capacity and mass use of RATs at points of care. Thus, the personnel at the emergency department may not have had the appropriate time and concentration to conduct the RAT according to manufacturer’s instructions. The discordance of the results of the two samplings from our study outlines the importance of following the procedures while using RAT and especially when mass testing is required.

## 5. Conclusions

Rapid Test Ag 2019-nCoV-PROGNOSIS, BIOTECH, which was used in the present study, exhibits a similar performance for SARS-CoV-2 detection for the B1.1.529 (Omicron) variant despite the high number of mutations (above 30) gathered in its viral genome. Consequently, the aforementioned RAT remains useful for COVID-19 diagnosis and mass screening. When an evaluation of the RAT is performed in clinical settings, the possibility that an overwhelming burden of work (as was observed during the epidemic wave from the Omicron variant) could affect the results must be carefully examined. Finally, if the mass use of RATs is required, laboratory expertise should be considered.

## Figures and Tables

**Table 1:** Specificity and sensitivity of the test according to Ct values of the disease.

		Sensitivity (%)				Specificity (%)			
Sampling Date		Positive Samples (n)		95% CI		Negative Samples (n)		95% CI	
March–April 2021	Total	141/165	85.5	79.1	90.5	458/459	99.8	98.8	100.0
30 December 2021	Total	66/139	47.5	39.0	56.1	75/80	93.8	86.0	97.9
	SGTF	51/111	46.0	36.5	55.7				
	Ct ≤ 25 (SGTF)	49/97	50.2	40.2	60.8				
5 January 2022	Total	77/98	78.6	69.1	86.2	53/53	100.0	93.3	100.0
	SGTF	71/90	78.9	69.0	86.8				
	Ct ≤ 25 (SGTF)	66/72	91.7	82.7	96.9				

Sensitivity, that is, the probability that a test result will be positive when the disease is present (true positive rate), was calculated for each group according to the following type: a/(a + b). Specificity, that is, the probability that a test result will be negative when the disease is not present (true negative rate), was calculated according to the following type: d/(c + d).

## Data Availability

Not applicable.
